# Effectiveness of psychological interventions for reducing depressive symptomatology and overload and improving quality of life in informal caregivers of non-institutionalized dependent elderly: a systematic review

**DOI:** 10.3389/fmed.2024.1394640

**Published:** 2024-06-19

**Authors:** Marco Antonio Barrero-Mejias, Sandra Gómez-Martínez, Jesús González-Moreno, María Rueda-Extremera, Eva Izquierdo-Sotorrio, María Cantero García

**Affiliations:** ^1^Faculty of Health Sciences and Education, Universidad a Distancia de Madrid (UDIMA), Madrid, Spain; ^2^Faculty of Health Sciences, Valencia International University, Valencia, Spain

**Keywords:** informal caregiver, depression, burden, quality of life, multiple component intervention

## Abstract

**Introduction:**

The phenomenon of aging is distinguished by profound life transformations, with the most dependent group being constituted by elderly individuals. The responsibility for their care primarily falls on the figure of the informal caregiver. The scarcity of time, the stress associated with caregiving, the financial, work-related, and personal difficulties it entails, make it a collective with high probabilities of experiencing various psychological disorders. Interventions that have shown the best results are those of multiple components, composed of various techniques that seek to adapt to the reality of the informal caregiver.

**Method:**

The purpose of this study is a systematic review of effective interventions on depressive symptoms, emotional wellbeing, burden, or quality of life in informal caregivers of non-institutionalized dependents from 2018 to the present. A search was conducted in November 2023, on Pubmed, Pubmed Central, Proquest, and Scielo. The final review was conducted on 11 articles.

**Results:**

The results indicate that multiple component interventions including cognitive behavioral techniques and psychoeducation in combination with stress coping techniques and social support are more effective on depressive symptoms, burden, quality of life, and increasing the social support network.

**Discussion:**

Results on web-based programs demonstrate their efficacy and effectiveness, but require a greater number of trials to adjust their methodological quality and content to the idiosyncrasies of the informal caregiver.

## 1 Introduction

Dependency refers to the chronic condition in which individuals, due to a variety of factors (such as age, illness, disability, etc.), have lost part or all of their physical, mental, and intellectual capacity and require assistance or care from others to perform all daily activities (1). There are several mechanisms to ensure adequate care for dependent individuals, but the burden of care still mainly falls within the family. This type of care, commonly referred to as informal care (IC), is characterized by being unpaid work, taking place in the home itself with limited resources, and involving a kinship relationship (2–4). IC for a dependent person causes a higher level of stress and anxiety than that of other chronic illnesses, being present in more than 75% of caregivers (5). More than 50% of informal caregivers (ICGs) exhibit depressive symptoms (DS) at a significant level, and as the caregiving time progresses, they may show higher probabilities of developing major depressive disorder (6–9). They must cope with more daily problems, suffer economic complications, lack leisure time, have limited social relationships, and express a greater sense of overall discomfort in their lives (7, 10–16). The characteristics of the dependent person’s illness correlate with the health of the ICG, their level of anxiety, and their quality of life (QoL), constituting one of the predictive variables for the development of DS (7, 16–19).

The “Caregiver Syndrome” is the most used concept to explain all the risk factors that affect the health of ICGs, in psychological, physical, social, and economic aspects, due to the care of dependent individuals, generating a high level of stress over a long period of time (20–22). Overload is the most studied concept in relation to the discomfort and deterioration of the QoL experienced by ICGs (23), with the variables that constitute it being related to the stressful situations faced by ICGs, dedication time, age, educational level, social status, socioeconomic factors, social support network (SSN), their own coping strategies, and the relationship and type of dependency of the care recipient (24–28), showing that, the higher the overload, the higher the risk of anxiety, depression, lower social support (SS), and longer caregiving time (3, 6, 14, 23, 25, 29–31). Therefore, the caregiver syndrome is not a simple concept to define due to its multidimensionality (32) and, consequently, to evaluate (28). Regarding the profile of the ICG, it is mostly women, middle-aged, married, with a low educational level, and closely related to the care recipient, usually daughter or spouse, with a caregiving time exceeding 8 h per day (3, 14, 30, 31, 33–35).

In line with this multidimensional model of IC, numerous interventions have been developed to mitigate the negative effects of this type of care, supported by numerous research studies that have shown significantly high results, among which psychoeducational interventions (PEIs), cognitive-behavioral interventions (CBT), multiple component interventions (MCIs), group support (GS), stress coping (SC), problem-solving (PS), and some third-generation therapies, such as behavioral activation therapy (BAT) and acceptance and commitment therapy (ACT), stand out. It has been evidenced that MCIs and CBTs are the ones that maintain the best results, especially in variables such as depression, and secondarily affecting variables such as burden, stress, and anxiety, improving the QoL and emotional wellbeing (EWB) of ICGs, thereby enhancing care (36–43). Interventions carried out in different modalities, especially by phone (IT) and online (IOL), show significant results, although with less efficacy than the interventions mentioned above, but they encourage further research on their effectiveness (44–47). The aim of this document is to conduct a review of the most recent studies on the effectiveness of interventions in reducing DS, burden, and improving the QoL of dependent individuals’ ICGs. It is hypothesized that MCIs, with techniques derived from CBT, are the most suitable for eliminating or reducing the DS of dependent individuals’ ICGs. A second hypothesis is proposed in which MCIs, with techniques based on psychoeducation and SS, improve QoL, reduce burden, and increase SSN.

## 2 Materials and methods

A systematic review of articles, in both English and Spanish, published between the years 2018 and 2023 has been conducted, following the guidelines established by the PRISMA protocol (48).

### 2.1 Eligibility criteria

For the search and selection of studies, the PICO(s) strategy has been employed (Table 1). The inclusion criteria for the selection of research have been as follows: (a) the intervention targets caregivers of elderly individuals who do not reside in specialized centers, (b) they must assess the EWB, burden, QoL, and DS of the ICG, (c) the evaluation must be conducted at least at two time points, (d) the intervention has a control group, and (e) participation in each intervention group must be controlled and randomized. The exclusion criteria for trials have been as follows: (a) the dependent individual is institutionalized, (b) the dependent individual is under 60 years old, (c) the intervention focuses on the dependent older adult, and (d) the intervention focuses on other psychological and medical aspects of the caregiver (Table 2).

**TABLE 1:** Process description PICO(s).

P	Informal caregivers, presenting mild or moderate symptoms of depression or emotional distress, of non-institutionalized dependent persons over 65 years of age.
I	Psychoeducational, cognitive-behavioral, problem solving, contextual therapies
C	Randomized controlled trial with a control or comparison group.
O	Quality of life, overload, social support network, depressive symptomatology.
(s)	Longitudinal study

**TABLE 2:** Selection criteria.

Inclusion criteria	Exclusion criteria
Caregivers of non-institutionalized elderly people	Institutionalization of the person receiving care
Variables of emotional distress, overload and depressive symptoms of the caregiver.	Dependent person under 60 years of age
Evaluation with two measurement moments	The dependent person receives the intervention
Existence of a control group and an intervention group.	Intervention in non-psychological aspects
Randomized controlled trial	

### 2.2 Sources and information search strategies

A search was conducted in the databases of “Proquest,” “Pubmed,” “Pubmed Central,” and “Scielo.” This search ended on 14 November 2023, with articles published after that date not considered. The following search terms were established both in Spanish (Intervención, cuidadores, Dementia, depressión, Alzheimer) and in English (Intervention, caregiver, dependent, Alzheimer, Dementia, quality of life, depression), using the Boolean operators “AND,” “NOT,” and “OR” (Table 3).

**TABLE 3:** Information sources and search strategies.

Database	Search equation	Number of references found
Pubmed	(Intervention*) AND (caregiver*) AND (dependent* OR Alzheimer* OR Dementia*) AND (Therapy*) AND (quality of life* OR depression*) Filters: 2018-present Randomized controlled trial Free full text	(71)
Proquest	“Intervención AND cuidadores AND Dementia AND depressión” Filters: Full text Sciences journals Articles Spanish and English 2018–2023	(46)
Scielo	(Intervention) AND (caregiver) AND (dependent OR Alzheimer OR Dementia) AND (quality of life OR depression) Filters: 2018-present	(10)
Pubmed Central	(Intervention [Title] AND Caregiver AND controlled trial [Title] NOT protocol [title]) AND (Alzheimer OR dementia) AND (depression) Filters: 2018–2023	(58)

*Represents wildcard characters.

### 2.3 Publication selection

**FIGURE 1:**
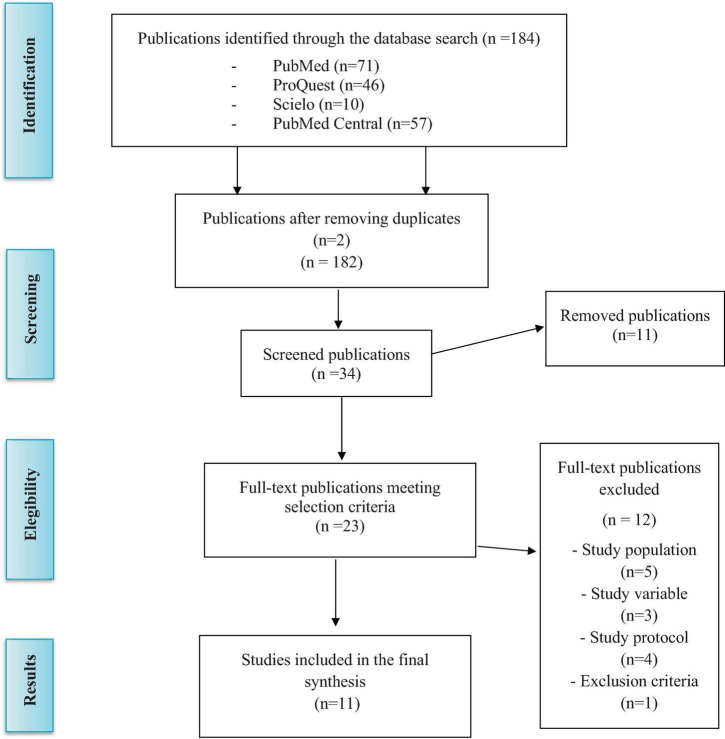
Flowchart based in Moher et al. (49).

Applying the information search strategy and once filters were applied using automatic tools from the meta-search engines (randomized controlled trial, open access, full text) and based on the criteria established previously, 184 articles were selected (*N* = 184) (Figure 1) (49). After reading the title and abstract, 23 articles were selected (*N* = 23). Once this set of articles was retrieved, they were read and analyzed accordingly, discarding those that did not meet the inclusion criteria (*N* = 12). For the final analysis of this review, 11 articles were included (*N* = 11).

### 2.3.1 Data analysis

To analyze the results of the selected primary studies, the following variables have been considered: (1) Sample size, age, educational level, and caregiving time to contrast results with other studies. (2) Evaluated variables such as DS, burden, QoL, and EWB. (3) Assessment instruments used to measure the aforementioned variables. (4) Type of intervention applied, both in the experimental group (EG) and in the control group (CG). (5) Analysis of methodological quality, analyzing those studies with inclusion and exclusion criteria, type of clinical trial, and if they had a CG. The analysis of methodological quality was carried out using the PEDro scale that evaluates internal validity through 10 criteria, leaving the first criterion out of the calculation as it is related to the external validity of the trial. (6) Intervention results and effects, analyzing statistically significant changes from EG compared to CG for the evaluated variables. (7) To assess the risk of bias presented by the selected studies with the aim of providing good evidence, the Cochrane Risk of Bias Assessment Tool proposed in the Cochrane Handbook for Systematic Reviews (2011) was used.

## 3 Results

### 3.1 Study characteristics

All selected studies are randomized controlled trials (RCTs), with random allocation to one or more EGs and one CG. The most evaluated variables in all RCTs are DS (*N* = 6), burden (*N* = 7), EWB (*N* = 5), QoL (*N* = 6), and SSN (*N* = 4), whose baseline measures, obtained through relevant assessment instruments, indicated acceptance of the sample for the proposed intervention in each of the trials (Table 4).

**TABLE 4:** Classification of interventions and study variables.

			Variables				
Intervention	Mod	ECA	SD	SOBC	SA	V	E
IMC	S	Cerquera-Córdoba et al. (52)					
	S	Birkenhäger-Gillesse et al. (53)					
	IOL	Teles et al. (54)					
	IT	Donath et al. (55)					
	S	Kor et al. (56)					
	IOL	Meichsner et al. (51)					
	IOL	Boots et al. (45)					
	IOL/IT	Possin et al. (50)					
AS	IOL	Christie et al. (57)					
	IOL	Dichter et al. (58)					
	IOL	Tinoco-Camarena et al. (59)					

IMC, multicomponent intervention; AS, social support intervention. Mod, modality; S, synchronous; IT, telephone intervention; IOL, online intervention; ECA, randomized controlled trial; SD, depressive symptoms; SOBC, overload; AS, social support; RSA, social support network; CV, quality of life; BE, emotional wellbeing.

### 3.2 Sample

Combining all selected studies, 1,743 participants constitute the total sample, of which 1,242 participants (72%) are female, with an average age of 60 years. In most studies, and for which data are provided, it is the daughter or son (55%) or spouse (40%) who is the primary ICG, with an average of 3.46 years of continuous caregiving. The sample size varies greatly among articles, with the largest sample size in the study by Possin et al. (50), with a sample of 780 participants, and the smallest in the study by Meichsner et al. (51), with a total of 37 participants. The educational level of the constituent sample varies from one study to another, highlighting that 54% of the sample has secondary education, followed by 35% with higher or tertiary education. The basic level is below 35% of the total.

### 3.3 Intervention

All RCTs are characterized by aiming to intervene on the effects of caregiving in ICGs of dependent individuals, but they differ in the study variables, from reducing DS to improving QoL or increasing SSN, and therefore, they differ in the type of intervention applied. Hence, studies are classified according to the type of intervention and specify whether they were applied in online or telephone modalities (Table 4).

### 3.4 Multiple component interventions

In the study by Cerquera-Córdoba et al. (52), the multicomponent program designed aims to decrease burden and increase SSN for the caregiver. It includes components of psychoeducation, systemic communication and physiotherapy, assertive communication, mental health, subjective wellbeing, caregiving skills, and SSN enhancement, both professional and functional. It is designed with a methodology composed of three intervention groups: Group A receives the MCI, Group B receives a respite program, and Group C or CG is on a waiting list. In the study by Birkenhäger-Gillesse et al. (53) titled “More at home with dementia,” the objective is to improve QoL, analyzing secondary outcomes such as burden and EWB (53). The multicomponent program includes educational components and CBT that address psychological and educational issues related to emotional, relational, practical, financial, and social changes associated with living with someone with dementia, as well as group work techniques, modeling, and role-playing delivered in group sessions. The CG receives a care relief intervention. Teles et al. (54) present in their study the online and self-guided program “iSupport,” a pilot test carried out in Portugal that requires cultural adaptation, with the aim of reducing burden, DS, anxiety, increasing positive caregiving aspects and self-efficacy, and improving QoL. Its components include education on caring for the dependent person and SS for caregivers using techniques such as PS, CBT, SC, or BAT. It is presented as a self-guided online application with five modules and 23 lessons. The CG receives an educational program.

Donath et al. (55) developed a psychoeducational telephone intervention (TI) on dementia and stress coping (ACE) tailored to the needs of caregivers with the aim of reducing caregiver burden and DS and improving QoL. The control group (CG) receives no intervention. They also intervene with people with dementia to verify if the effects of the intervention are related to the cognitive decline of the care recipient. Kor et al. (56) relied on CBT combined with Mindfulness to develop their intervention with the aim of reducing perceived stress as the primary outcome and burden, QoL, depression, and anxiety as secondary outcomes. The program included psychoeducation on stress and caregiving for the dependent person and mindfulness activities with components such as meditation and mindful eating, understanding unpleasant feelings, and awareness of difficult feelings, thoughts, and sensations related to caregiving. The CG receives standard care plus an educational session on dementia caregiving. The online program “Partner in Balance (PiB)” with face-to-face support, described in the study by Boots et al. (45), includes components such as acceptance, communication with family and social environment, and stress management techniques, focusing on the positive, insecurities, reflections, self-awareness, social relationships, and support. Its aim is to improve self-efficacy and mastery of ICGs in early stages of caregiving and also to evaluate its impact on DS, QoL, anxiety, and stress. The CG is on a waiting list.

Possin et al. (50) present an Internet-based intervention combined with TI called “Care Ecosystem,” composed of supportive care for dementia caregivers with the aim of reducing burden and DS and thereby improving the QoL of the care recipient. The team first addresses the immediate needs of ICGs, then identifies common problems and provides personalized support and education, following a standardized care plan. Both the care recipient and the caregiver are the recipients of the program. Outside the program, a team handles medical needs, problematic behavioral symptoms, legal or financial circumstances. The CG receives general articles related to dementia.

The study by Meichsner et al. (51) describes a trial using an Internet-based intervention, the Tele.TAnDem program, which customizes the intervention according to the specific needs of each caregiver, employing mixed techniques of Acceptance and Commitment Therapy (ACT) and CBT. It was conducted online with the aim of supporting ICGs in a more flexible manner. It consists of 10 modules aimed at reducing caregiver DS and enhancing their psychosocial resources and improving their EWB. At the beginning of the intervention, platform users set their personal goals, and the intervention is designed based on the assessment of those goals. The control group (CG) is on a waiting list.

### 3.5 Social support interventions

The online program “Inlife,” described in Christie et al. (57), aims to provide SS and encourage the development of positive interactions with loved ones, friends, and other significant members of their support network, analyzing secondarily its effect on loneliness, psychological distress, and QoL.

Dichter et al. (58) developed the Internet-based intervention “Talking Time.” This intervention is based on support groups where the exchange and reciprocal learning are important and how these can influence emotional wellbeing, QoL, and other psychological variables. The CG remains on a waiting list.

Tinoco-Camarena et al. (59) present an online program called “Dialog Circles” to reduce burden and improve positive mental health, conducted by a team of nurses, with a duration of 90 min and a frequency of 15 days in which SS is provided by creating safe spaces that allow participants, organized in groups, to express problems, difficulties, and feelings about their role as caregivers.

All studies have had at least two measurement points. Here, T0 will be defined as the pre-test or initial evaluation moment, T1 as the post-test moment, T2 as the follow-up, and T3 as a third moment or a second follow-up, if any. Thus, in the selected trials, we find that all have a T0 and T1, with an average time of 3 months from the start of the study. Nine of the studies have a T2 with an average of 6.2 months from the start of the trial. The RCTs by Boots et al. (45) and Dichter et al. (58) are the only ones that do not have a T2.

#### 3.5.1 Results of methodological quality

The results of methodological quality measured through the PEDro Scale (Table 5) show that only one study reaches excellent methodological quality with 10 points (56). The rest of the studies demonstrate good methodological quality with scores ranging from 6 to 8 points (45, 50, 51, 53–59). In the RCTs where subjects and therapists could not be blinded, it is specified that blinding could not be achieved due to the type of intervention applied, mostly because it was a web-based or telephone-based intervention. Overall, methodological quality is good in the selected studies.

**TABLE 5:** Methodological quality results.

Escala PEDro												
**References**	**1**	**2**	**3**	**4**	**5**	**6**	**7**	**8**	**9**	**10**	**11**	**Puntuación total**
Cerquera-Córdoba et al. (52)	Y	Y	Y	Y	N	N	Y	Y	Y	Y	Y	8
Meichsner et al. (51)	Y	Y	Y	–	Y	N	N	N	Y	Y	Y	6
Boots et al. (45)	Y	Y	Y	Y	N	–	Y	Y	Y	Y	Y	8
Possin et al. (50)	Y	Y	Y	Y	N	–	Y	Y	Y	Y	Y	8
Birkenhäger-Gillesse et al. (53)	Y	Y	Y	N	N	N	Y	N	Y	Y	Y	6
Dichter et al. (58)	Y	Y	Y	N	Y	N	Y	Y	Y	Y	Y	8
Kor et al. (56)	Y	Y	Y	Y	Y	Y	Y	Y	Y	Y	Y	10
Teles et al. (54)	Y	Y	N	N	N	Y	Y	N	Y	Y	Y	6
Tinoco-Camarena et al. (59)	Y	Y	Y	–	Y	Y	–	Y	Y	Y	Y	8
Donath et al. (55)	Y	Y	Y	–	N	N	Y	N	Y	Y	Y	6
Christie et al. (57)	Y	Y	Y	N	–	–	–	Y	Y	Y	Y	6

1. Choice criteria were specified; 2. Subjects were randomly assigned to groups; 3. Allocation was concealed; 4. Groups were similar at baseline with respect to the most important prognostic indicators; 5. All subjects were blinded; 6. All therapists administering therapy were blinded; 7. All assessors measuring at least one key outcome were blinded; 8. Measures of at least one of the key outcomes were obtained from more than 85% of subjects initially assigned to groups; 9. Results were presented for all subjects who received treatment or were assigned to the control group, or when this could not be, data for at least one key outcome were analyzed by “intention-to-treat”; 10. Results of statistical comparisons between groups were reported for at least one key outcome. 11. The study provides point and variability measures for at least one key outcome. Y: Yes. N: No.

### 3.6 Results of bias risk

Table 5 presents the results of the Cochrane bias risk assessment, and each of the evaluated items is described below. All selected RCTs show a low risk in random sequence generation. The methods used include computer generation, block allocation, allocation by hour and date of submission, and random selection.

### 3.7 Allocation concealment

All RCTs show a low risk in this criterion. All subjects were recruited through various means, including mailing lists, social networks, advertising, community centers, associations, etc., for subsequent evaluation and random group allocation, preventing prediction of group assignment.

### 3.8 Blinding of participants and personnel

Only two studies (56, 59) demonstrated adequate blinding of participants and personnel, presenting a low risk of bias. In the remaining articles, except for Birkenhäger-Gillesse et al. (53) and Christie et al. (57), where masking is not clear, there is a high risk of bias due to no concealment of the type of intervention carried out for both participants and personnel. In all cases, the recipient of the intervention knows which group they belong to, mainly due to the type of intervention received.

### 3.9 Blinding of outcome assessors

All RCTs show a low risk of bias in outcome assessment, as they include trials in which the assessors were blinded at all measurement points, either due to unawareness of group allocation or through the design of computerized self-assessment. Only four RCTs show a low risk of bias in this criterion (51, 52, 55, 59). In these trials, results regarding participant dropout or attrition are analyzed, demonstrating a low dropout rate that does not affect the displayed results, and effects are analyzed based on initial measurements. In the remaining studies, methods such as intention-to-treat analysis or protocol approaches are employed to address missing data in the study. In the trials by Tinoco-Camarena et al. (59) and Christie et al. (57), the provided data are insufficient to identify the risk of bias in the displayed programs.

All RCTs have study protocols or rely on previous studies with minimal or no changes, presenting results based on them and reporting expected and anticipated outcomes. Despite Donath et al.’s (55) trial having a protocol, it is the only one that presents a high risk of bias, as it focused solely on results that were significant in the primary analysis.

The variability in the scales and measurement instruments used in each study and the disparity in their content, along with the particular characteristics of the sample, can also represent a bias risk to consider.

### 3.10 Intervention effects

The results presented below are categorized according to the therapeutic objective of the intervention (Table 6), considering significant differences between groups at the time of measurement (*p* < 0.05) and the intervention effect size (if data are provided).

**TABLE 6:** Results of the interventions.

References	Sample age dropouts % (GE-GC)	Variables evaluated	Measuring instruments	Type of intervention	Intervention groups (Design)	Duration/ format/ No. of sessions/ measurement moments	Results
Cerquera-Córdoba et al. (52)	N: 58 H: 12 M: 38 55.1 years 0–16–45%	Burden Social support network	ZBI MOS	IMC: Psychoeducation, communication, systemic social support network, and physiotherapy	EG1 = IMC+ respite EG2 = IMC CG = Waiting list	Once a week/4 h per day Synchronous T0; T1 (5 months);T2 (10 months)	Burden reduction: T1 = 13.1 (*p* = 0.001); T2 = 15.4 (*p* = 0.001). Increase RSA: T1 = 10.8 (*p* = 0.09); T2 = 13.2 (*p* = 0.039).
Meichsner et al. (51)	N: 37 H: 8 M: 29 62.11 years 16.2–10.8%	Depression, Psychosocial resources Emotional wellbeing	CES-D CGS PRUQ	Tele.TAnDem: TCC	EG = Access to platform GC = Waiting list	10 modules (11.4 weeks) Online T0; T1 (8 weeks); T2 (5 months)	Depression T1 (*p* = 0.910; *d* = 0.076). T2 (*p* = 0.244; *d* = 0.682) RSA T1 (*p* = 0.029; *d* = 0.750) T2 (*p* = 0.561; *d* = 0.190). BE (*p* = 0.023)
Boots et al. (45)	N: 81 H: 28 M: 53 69 years 8.1–2.43%	Self-efficacy, depression, stress, quality of life	CESS PMS HADS-A CES-D	Partner in Balance: Acceptance, communication, stress management, social support network	EG: Access to platform GI: Waiting list	4 modules, two weeks por module. Online T0; T1 (8 weeks)	EG > CG Self-efficacy: (*p* = 0.395) Mastery: (*p* = 0.001; *d* = 0.94) CV: (*p* = 0.032; *d* = 0.58) No significant differences EG-CG: Depression: (*p* = 0.293) Stress: (*p* = 0.374)
Possin et al. (50)	N: 780 H: 227 M: 553 64.7 years 13–15%	Depression, burden, self-efficacy	PHQ-9 ZBI	Care Ecosystem (IOL). IMC: Social support and psychoeducation.	EG = Access to platform CG = Care information	7 modules Online T0; T1 (6 months); T2 (12 months)	EG > CG Depression in T2 (*p* = 0.03). Autoeficacia T1 (*p* = 0.001) but no in T2 (*p* = 0.11). Burden T2 (*p* = 0.046) Satisfacción con la IOL: 45.5%
Birkenhäger-Gillesse et al. (53)	N: 108 H: 33 M: 75 73 years 26–38%	Quality of life, burden, health, emotional wellbeing	CES-D HADS-A CarerQol-7D SF-36 EQ-5D-3L	IMC: Psychoeducation, modeling, role-playing, psychotherapy	EG: IMC CG: Care relief	5 days in 14 group sessions in 16 groups T0; T1 (3 months); T2 (6 months)	No significant differences : CV (*p* = 0.99), Burden (*p* = 0.71) BE (*p* = 0.13) Less participation than expected and high dropout rate
Dichter et al. (58)	N: 38 H: 6 M: 32 65.5 years 5–5%	Quality of life, emotional and psychological wellbeing, social network	SF-12 PSSC-9 HRQoL	Talking Time: Social support groups	EG: Talking-time CG: Usual care	1 h every 2 weeks, 4 modules, Phone and face-to-face. T0; T1 (3 months)	No significant results for any variables. Small effect size in health-related QoL (5.77) Small effect size in AS (0.43)
Kor et al. (56)	N: 113 H: 35 M: 78 61.7 years 8.9–7%	Stress, depression, caregiver distress, quality of life	PSS ZBI CES-D HADS-A SF-12 FFMQ-SF	IMC: Modified Mindfulness-Based Cognitive Therapy (MBCT) for dementia care	EG: MBCT CG: Usual care + education	7 sesiones, 2 h/sesión, 10 weeks Synchronous T0; T1 (10 weeks); T2 (6 months)	EG < CG. Significant differences in T1 and T2: Stress: Stress (*p* = 0.02 and *p* = 0.03) Depression (*p* = 0.001 and *p* = 0.04; *d* = 0.9) Mental health-related QoL at T2 (*p* = 0.001; *d* = 0.6)
Teles et al. (54)	N: 42 H: 10 M: 32 53.55 years 48–5%	Caregiver burden, depression, quality of life	ZBI HADS-A PAC GSE HOQOL-BREF	iSupport: IMC: TCC, TSP, AC	EG: IMC CG: Educational program	5 modules Online T0 T1 (3 months) T2 (6 months)	No significant differences in: Caregiver burden (*p* = 0.800; *x*^2^ = 0.45) Depression (*p* = 0.347; *x*^2^ = 1.31) CV (*p* = 0.973; *x*^2^ = 0.06) Significant differences in: Environment-related QoL (*p* = 0.029; *x*^2^ = 7.06)
Tinoco-Camarena et al. (59)	N: 86 H: 17 M: 69 56 years 0%	Caregiver burden, positive mental health	ZBI-7 PMHQ	Dialog Circles. Group social support	GE = Self-help group GC = Usual care	3 sessions of 90 min, every 15 days, Group, Online T0; T1 (7 months)	Caregiver burden T0 (*p* = 0.01); T1 (*p* = 0.01) Positive mental health: T0 (*p* = 0.01); T1 (*p* = 0.01)
Donath et al. (55)	N: 359 H: 77 M: 227 59.5 years 15.1–14.9%	Burden, depression	BSFC-S OMS-5 WHO-5	IMC: Dementia psychoeducation and management of challenging behaviors, Stress coping	EG: IMC CG: Usual care	60 min., 1 session/week, Phone T0; T1 (6 months); T2 (6 months)	Significant differences in caregiver burden EG > CG. T1 (*t* = 2.10; *p* = 0.037). T2, (*t* = 2.35; *p* = 0.019; *d* = 0.18) Depression no significant differences (*p* = 0.089). (*d* = 0.177; *t* = 1.49)
Christie et al. (57)	*N* = 96 H: 31 M: 65 56.9 years 17–6.25%	Perceived social support, psychological complaints, quality of life	SSCQ MSPSS LSNS-6 HADS PSS ICECAP	Inlife. Social Support and Positive Interactions Strengthening program	EG: Inlife CG: Waiting list	16 weeks Online Flexible acces T0; T1 (8 weeks); T2 (16 weeks)	No significant differences between groups or over time: Social support: (*p* = 0.62), Anxiety and Depression (*p* = 0.31), CV (*p* = 0.93) The intervention may have influenced awareness of the lack of social support.

No., number; N, sample; M, female gender; H, male gender; IMC, multicomponent intervention; IOL, online intervention; EG, experimental group; CG, control group; RSA, social support network; CBT, cognitive behavioral therapy; SD, depressive symptoms; BE, emotional wellbeing; ZBI, Zarit Overload Scale; MOSS, medical social support disengagement study questionnaire; CES-D, Center of Epidemiological Studies Depression Scale; CGS, The Caregiver Grief Scale; PRUQ, psychosocial resource utilization questionnaire for family caregivers of people with dementia; CV, quality of life; CESS, Caregiver Self-Efficacy Scale; PMS, Pearlin Mastery Scale; HADS-A, A Hospital Anxiety and Depression Scale-Anxiety; PHQ-9, Patient Health Questionnaire; CarerQol-7D, The Care-related quality of life instrument; SF-12; SF-36, the short form-12-36 health survey; EQ-5D, EuroQol Cuestionarie; HRQoL, health-related quality of life; PSSC-9, perceived social support caregiving; PSS, Perceived Stress Scale; FFMQ-SF, five facets mindfulness; IMC, multi-component intervention; TSP, problem-solving therapy; AC, behavioral activation therapy; PAC, positive aspects of caregiving; GSE, Generalized Self-efficacy Scale; HOQOL-BREF, quality of life scale; PMHQ, Positive Mental Health Questionnaire; BSFC-s, Brief Family Caregiver Overload Scale; OMS-5, wellbeing index; WHO-5, health-related quality of life or perceived health; SSCQ, short sense of competence questionnaire; MSPSS, the multidimensional scale of perceived social support; LSN-6, The Lubben-6 Social Network Scale; PSS, Perceived Stress Scale; ICECAP, The ICEpop CAPability measure for adults.

### 3.11 Depressive symptoms

Meichsner et al. (51) demonstrated that the IOL “Tele.TAnDem,” based on CBT, did not have significant effect sizes on depressive symptoms (*d* = 0.076), nor were there significant differences between group experimental (GE) and group control (GC) (*p* = 0.910) at T1. However, in the measurements at T2, the intervention had a medium effect size, although there were no significant differences between GC and GE (*d* = 0.682; *p* = 0.244), with GE showing a decrease in symptom scores over time, while in GC they had slightly increased, concluding that the intervention effect was somewhat insignificant. In the “Partner in Balance (PiB)” program by Boots et al. (45), depressive symptoms did not show a significant effect (*f* = 1.13), and there were no differences between GC and GE (*p* = 0.293). There was a difference in the self-efficacy domain (*p* = 0.395), and it was expected that increasing self-efficacy (attention management, service utilization, care mastery) would decrease depressive symptoms and burden, but this was not observed in the results. One possible explanation provided by the authors is that the program targeted a population of caregivers in the early stages of care, characterized by less stressful situations. Possin et al. (50), in their combined IOL with IT, a multicomponent type, “Care Ecosystem,” showed significant results for GE in depressive symptoms at T2 (*p* = 0.03). The treatment effect at T1 was also statistically significant (*p* = 0.001). Furthermore, in the GE, the number of caregivers with high depression scores decreased from 49 to 29 participants by the end of T2 (*p* = 0.004), while in the GC, it slightly increased from 16 to 22 individuals (*p* = 0.22). These positive results were a result of individualized attention to caregivers along with addressing their most immediate needs, in addition to more individualized specialist phone contacts. Kor et al. (56), in their combined TCC and Mindfulness program, showed significant differences between GE and GC in depressive symptoms in favor of the treatment group (T1: *p* = 0.001; T2: *p* = 0.04), with a large effect size (*d* = 0.90). The combination of both techniques seems to have had a positive effect on re-evaluating negative thoughts, influencing a decrease in depressive symptoms. Teles et al. (54) presented the multicomponent intervention “iSupport,” with no significant differences for depressive symptoms (*p* = 0.347), although there were for anxiety. Donath et al. (55), in their telephone-based multicomponent intervention, with psychoeducational and ACE components, report that, for depressive symptoms, there are no significant differences between GE and GC over time (*p* = 0.089). During the intervention, depressive symptoms decreased but increased at T2. The results show that there is an effect on depressive symptoms during the intervention period, but these effects do not persist over time. Finally, the “Inlife” study developed by Christie et al. (57), on social support, in online mode, one of the secondary outcomes evaluated was depression, with a somewhat insignificant difference between groups (*p* = 0.31) at T2. The intervention had no effect on depressive symptoms. Its main components were aimed at improving caregivers’ social support, and there was no significant interaction between this variable and depressive symptoms.

### 3.12 Burden

In the study by Cerquera-Córdoba et al. (52), significant differences at T1 and T2 regarding burden (*p* = 0.001) were attributed to psychosocial support aimed at providing strategies for the empowerment of the primary caregiver and group interventions aimed at improving self-care and, consequently, the care of the dependent person. The GE, in its initial evaluation, showed higher results in the burden variable, so the effect size calculation considered that initial measurement rather than the comparison between groups. The multicomponent intervention proposed by Birkenhäger-Guillese et al. (53) does not show improvement in burden (*p* = 0.71), although it does show significant differences in the subscale assessing physical and emotional function using the SF-36 scale (*p* = 0.01). In the trial by Possin et al. (50), burden decreased more in the GE than in the GC at T1 (*p* = 0.008) and T2 (*p* = 0.046). These results were attributed to the psychoeducational component, which reduced the need for external attention (visits to health centers, emergencies, hospitalizations, etc.), thus reducing the burden of care as caregivers had more information and support. In the trial by Teles et al. (54), there were no effects on burden (*x*^2^ = 0.45) or significant differences between GC and GC (*p* = 0.80). These results were difficult to assess in the intervention context, as during the pandemic lockdown, platform participants could not leave their homes, increasing caregiver stress. The authors consider these variables for future large-scale program trials. Tinoco-Camarena et al. (59), in the online self-help group intervention, show statistically significant results in burden at both T1 and T2 (*p* = 0.01). The support received through the intervention, as well as the information provided by the self-help group, combined with very high participation and no dropout rate, showed that the intervention influenced lower burden and, furthermore, correlated with self-care and improvement in the care received by the dependent person. In the trial presented by Kor et al. (56), stress decreased (*p* = 0.03), but not physical burden at T2 (*p* = 0.39). On the other hand, in the trial by Donath et al. (55), burden did not show a significant difference for both groups (*p* = 0.126), with a small effect size (*d* = 0.20). However, regarding time, the GE showed an improvement in burden compared to the GC (T1, *p* = 0.037; T2, *p* = 0.019). In general, all presented trials show an improvement in the burden variable, especially in multicomponent interventions that include specific techniques to reduce primary caregiver burden, based on psychoeducation and social support.

### 3.13 Social support

In the study by Cerquera-Córdoba et al. (52), social support availability (SSA) is increased by the intervention in the GE, with a statistically significant difference between initial and post-treatment measures (*p* = 0.09), maintaining its effect over time (*p* = 0.039). Having time, during which care was provided by another person for certain hours, as well as obtaining self-help strategies, allowed primary caregivers to establish new social relationships and more time to participate in the intervention program. The study by Meichesner et al. (51) evaluated the impact of the intervention on the use of psychosocial resources, finding no significant differences between groups at T1 (*p* = 0.781; *d* = 0.22) or at T2 (*p* = 0.750; *d* = 0.12). However, there were differences and intervention effects on the use of resources for emotional wellbeing (EWB) only at T1 (*p* = 0.023; *d* = 0.190). The telephone-based social support program “Talking-Time,” described in the study by Dichter et al. (58), had no significant effect on the social support variable between groups (*p* = 0.12), with a small effect size (*d* = 0.43). Similarly, the article by Christie et al. (57) presents an online intervention aimed at providing and enhancing social support, finding no significant difference in favor of the intervention between groups (*p* = 0.11). The authors suggest that the results were influenced by the limited number of interactions on the platform.

### 3.14 Quality of life

The study by Boots et al. (45) assessed the intervention’s effect on the QoL of the GE, finding a significant difference (*p* = 0.032), with a moderate effect size (*d* = 0.58). The trial by Birkenhäger-Gillese et al. (53) did not show significant differences in the QoL of the caregiver for the effects of the intervention (*p* = 0.99), measured through the CarerQol-7D scale. In the study by Kor et al. (56), a significantly greater improvement in mental health-related QoL was demonstrated at T2 compared to the GC (*p* = 0.001), with a large effect size (*d* = 0.6), not so for QoL related to physical health (*p* = 0.30). Teles et al. (54) did not show a significant difference between groups in overall QoL (*p* = 0.973), with a small effect size (*x*^2^ = 0.06) but did so in QoL related to the environment (*p* = 0.029) with a large effect size (*x* = 7.06). In the study by Christie et al. (57), it is detailed that there is no significant difference in the GE compared to the GC or over time. The study by Teles et al. (54) evaluated the overall QoL and environmental QoL of the groups, finding no significant differences in the former (*p* = 0.973) with a small effect (*x*^2^ = 0.06), but did so in the latter (*p* = 0.029) with a large effect (*x* = 7.06). Only the interventions show positive results on QoL, except for the trial by Birkenhäger-Guillesse et al. (53), where participants’ age and educational level could correlate with the results obtained, showing that younger participants and those with a higher educational level benefited more from this type of intervention, but needed it less compared to participants with a lower educational level and older average age.

### 3.15 Emotional wellbeing

The variable BE only showed significant differences in two programs: the multicomponent program by Meichsner et al. (51) and the AS program by Tinoco-Camarena et al. (59). In the first program, the component of writing down feelings and emotions of the caregivers through the platform, along with therapist guidance and feedback, increased the BE (*p* = 0.023). In the second program, the perceived support and self-help from the group led to an increase in BE (*p* = 0.001). You may insert up to 5 heading levels into your manuscript as can be seen in “Styles” tab of this template. These formatting styles are meant as a guide, as long as the heading levels are clear, Frontiers style will be applied during typesetting.

## 4 Discussion

Following the conducted review, it can be observed that, across all studies, the gender of the informal caregivers (ICs) remains predominantly female, middle-aged, daughters (> 50%) or spouses (> 40%), with varying educational levels from one study to another, indicating an increasingly higher level of education among ICs. Due to differences in the sociodemographic characteristics of the population across studies, it’s important to consider the context for conducting a detailed analysis from a gender perspective (60). The analysis confirms that interventions with multiple components (IMCs) are the most utilized within the established timeframe (2018–2023), albeit with differences in the intervened variables. Except for five trials (52, 53, 57–59), all aim to evaluate the effectiveness of interventions in reducing depressive symptoms (DS). The most common components in all these trials are psychoeducational, ACE, cognitive behavioral therapy (CBT), and social support (SS) interventions, which, in turn, have the most significant effect on the variable of caregiver burden and improvement of quality of life (QoL). These results are supported by other studies where psychoeducation and the application of ACE and SS techniques have a positive effect on caregiver burden and the stress associated with caregiving, improving the QoL of the IC (39, 61–63).

However, upon analyzing the components of each program, those utilizing CBT techniques show greater effectiveness and a significant difference in the intervention effect on the IC group in terms of depression and emotional wellbeing (BE). This is even more pronounced when combined with third-generation therapies like Mindfulness (56). or Acceptance and Commitment Therapy (ACT) Cheng et al. (64) concluded that psychoeducational programs along with mindfulness-based interventions were more effective in reducing DS than other IMCs, but younger individuals benefit more from mindfulness techniques, necessitating their combination with other techniques to address caregiver issues. Given that these are multicomponent programs, the combination of all their components has a positive effect on all evaluated variables. This conclusion suggests that caregiver burden is a multidimensional concept (32, 65), hence requiring an intervention adapted to each specific variable to be addressed. Kwon et al. (66) concluded that an effective tool for reducing DS lies in CBT. The same authors detailed that multicomponent programs including psychoeducation, SS, and CBT have positive effects on the studied sample. Similarly, Bustillo et al. (67) and Gallagher-Thompson and Coon (38) concluded that CBT-based interventions in combination with SS techniques and coping strategies or a combination of at least two theoretical approaches (41) show the best results, in line with Kor et al.’s trial (56), which combines CBT with Mindfulness, showing a large effect on DS, similar to the results of other reviews (68). A special mention to the “iSupport” program (54), which adapts to the cultural context of the intervention, obtaining good results in QoL, though not in DS, according to the authors themselves, due to the timing of the intervention, coinciding with the COVID pandemic.

Therefore, multicomponent programs combining psychoeducation with CBT-based techniques or contextual therapies like ACT or Mindfulness show better results in improving the BE and psychological wellbeing of ICs, enhancing their QoL. However, programs including psychoeducation along with SS techniques achieve greater benefit in obtaining social resources and reducing caregiver burden, as had been shown by previous studies (36–38, 69, 70). As observed in the results of the study by Meichsner et al. (51), CBT alone has a significant effect when intervening in the BE of the IC, with a significant effect over time, supported by other conducted studies, whose results were very similar (18, 26, 37, 40). However, unlike the aforementioned cited studies, the trial by Meichsner et al. (51) does not show significant efficacy on DS, mainly influenced by the content included in the platform, which mainly focused on emotional wellbeing related to anticipatory grief, rather than intervening in DS. Additionally, the intervention did not provide a structured approach by therapists that was similar for all participants, as flexibility was attempted to be provided in platform usage, along with the few exchanges within it between users and therapists.

Among the analyzed randomized controlled trials (RCTs), only the study by Tinoco-Camarena et al. (59) shows high efficacy of an intervention based on SS and self-help groups in variables such as caregiver burden and SS, while the studies by Christie et al. (57) and Dichter et al. (58) provide insignificant results on the same variables. These results are inconsistent with other studies, concluding that perceived SS of the caregiver increases thanks to self-help groups and interventions based on providing psychosocial support through professionals and shared experiences with other caregivers (71, 72). In the study by Christie et al. (57), it is noted that low involvement and commitment in the use of the IOL could explain the low efficacy both in the perceived SS and in the increase of the appraisal of social support (RSA). Furthermore, it is concluded.

### 4.1 Limitations and strengths

The number of trials, while not limited, is insufficient to fully grasp the effectiveness of the interventions due to the multitude of variables that must be considered, hence, it is recommended to conduct more comprehensive and separate studies to better understand intervention efficacy. Additionally, the number of randomized controlled trials (RCTs) found is scarce, given the broad search for effective interventions in informal caregivers (ICs) of dependent individuals. It would be interesting to carry out a more focused search on the effectiveness of more specific interventions. Lastly, the selected trials were conducted within the last 5 years, limiting the results. Therefore, it is recommended to consider different existing protocols for future reviews.

This work also has strengths. The inclusion of web-based programs provides interesting data to establish a new line of research that enhances program content for efficacy, effectiveness, and methodological quality improvement. Additionally, this review demonstrates that interventions with multiple components (IMCs) are the most applied in recent years based on studies conducted in the selected population, so continuing along this path could be positive for establishing solid interventions with greater efficacy in addressing the issues of ICs.

## 5 Conclusion

– IMCs combining psychoeducation and SS increase psychosocial resources and decrease caregiver burden.
– IMCs combining cognitive behavioral therapy (CBT) with other techniques are more effective in reducing Depressive Symptoms (DS) and increasing emotional wellbeing (BE).
– Internet-based interventions can improve the quality of life (QoL) and BE of ICs if they include various components combining different intervention models and tailored to the beneficiaries’ specificities.

More studies on the effectiveness and efficacy of Internet-based interventions are needed, as well as a more thorough study on the quality of interventions based on this modality.

## Data availability statement

The raw data supporting the conclusions of this article will be made available by the authors, without undue reservation.

## Author contributions

MB-J: Conceptualization, Data curation, Formal analysis, Funding acquisition, Investigation, Methodology, Project administration, Resources, Software, Supervision, Validation, Visualization, Writing – original draft, Writing – review and editing. SG-M: Conceptualization, Data curation, Formal analysis, Funding acquisition, Investigation, Methodology, Project administration, Resources, Software, Supervision, Validation, Visualization, Writing – original draft, Writing – review and editing. JG-M: Conceptualization, Data curation, Formal analysis, Funding acquisition, Investigation, Methodology, Project administration, Resources, Software, Supervision, Validation, Visualization, Writing – original draft, Writing – review and editing. MR-E: Conceptualization, Data curation, Formal analysis, Funding acquisition, Investigation, Methodology, Project administration, Resources, Software, Supervision, Validation, Visualization, Writing – original draft, Writing – review and editing. EI-S: Conceptualization, Data curation, Formal analysis, Funding acquisition, Investigation, Methodology, Project administration, Resources, Software, Supervision, Validation, Visualization, Writing – original draft, Writing – review and editing. MC: Conceptualization, Data curation, Formal analysis, Funding acquisition, Investigation, Methodology, Project administration, Resources, Software, Supervision, Validation, Visualization, Writing – original draft, Writing – review and editing.

## References

[1] Agencia Estatal Boletín Oficial del Estado. *Ley 39/2006, de 14 de diciembre, de promoción de la autonomía personal y atención a las personas en situación de dependencia (SAAD).* Marid: Boletín Oficial del Estado (2006). p. 299.

[2] García-Calvente MDM Mateo-Rodríguez I Eguiguren AP. El sistema informal de cuidados en clave de desigualdad. *Gac Sanit.* (2004) 18:132–9. 15171869 10.1157/13062262

[3] Cerquera Córdoba AM Galvis Aparicio MJ. Efectos de cuidar personas con Alzheimer: Un estudio sobre cuidadores formales e informales. *Pensam Psicol.* (2014) 12:144. 10.11144/javerianacali.ppsi12-1.ecpa

[4] Theng B Tran JT Serag H Raji M Tzeng H-M Shih M Understanding caregiver challenges: A comprehensive exploration of available resources to alleviate caregiving burdens. *Cureus.* (2023) 15:e43052. 10.7759/cureus.43052 37680399 PMC10480575

[5] Villarejo Galende A Eimil Ortiz M Llamas Velasco S Llanero Luque M López de Silanes de Miguel C Prieto Jurczynska C. Informe de la fundación del cerebro. Impacto social de la enfermedad de alzheimer y otras demencias. *Neurologia.* (2021) 36:39–49. 10.1016/j.nrl.2017.10.005 29249303

[6] Covinsky KE Newcomer R Fox P Wood J Sands L Dane K Patient and caregiver characteristics associated with depression in caregivers of patients with dementia. *J Gen Intern Med.* (2003) 18:1006–14. 10.1111/j.1525-1497.2003.30103.x 14687259 PMC1494966

[7] Mahoney R Regan C Katona C Livingston G. Anxiety and depression in family caregivers of people with Alzheimer disease: The LASER-AD study. *Am J Geriatr Psychiatry.* (2005) 13:795–801. 10.1097/00019442-200509000-00008 16166409

[8] Torres Á Blanco V Vázquez FL Díaz O Otero P Hermida E. Prevalence of major depressive episodes in non-professional caregivers. *Psychiatry Res.* (2015) 226:333–9. 10.1016/j.psychres.2014.12.066 25667119

[9] Pinyopornpanish K Soontornpun A Wongpakaran T Wongpakaran N Tanprawate S Pinyopornpanish K Impact of behavioral and psychological symptoms of Alzheimer’s disease on caregiver outcomes. *Sci Rep.* (2022) 12:1–9. 10.1038/s41598-022-18470-8 35986203 PMC9391353

[10] Vitaliano PP Zhang J Scanlan JM. Is caregiving hazardous to one’s physical health? A meta-analysis. *Psychol Bull.* (2003) 129:946–72. 10.1037/0033-2909.129.6.946 14599289

[11] Instituto de Mayores y Servicios Sociales. *Cuidados a las personas mayores en los hogares españoles.* Madrid: El entorno familiar (2005).

[12] Pinquart M Sörensen S. Helping caregivers of persons with dementia: Which interventions work and how large are their effects? *Int Psychogeriatr.* (2006) 18:577–95. 10.1017/s1041610206003462 16686964

[13] Deborah Majerovitz S. Predictors of burden and depression among nursing home family caregivers. *Aging Ment Health.* (2007) 11:323–9. 10.1080/13607860600963380 17558583

[14] Flores GE Rivas RE Seguel PF. Nivel DE sobrecarga en El desempeño Del Rol Del cuidador familiar DE adulto mayor con dependencia Severa. *Cienc Enferm.* (2012) 18:29–41. 10.4067/s0717-95532012000100004 27315006

[15] Espín Andrade AM. Factores de riesgo de carga en cuidadores informales de adultos mayores con demencia. *Rev Cubana Salud Publica.* (2012) 38:393–402.

[16] Watson B Tatangelo G McCabe M. Depression and anxiety among partner and offspring carers of people with dementia: A systematic review. *Gerontologist.* (2018) 59:e597–610. 10.1093/geront/gny049 29878117

[17] Pérez Peñaranda A García Ortiz L Rodríguez Sánchez E Losada Baltar A Porras Santos N Gómez Marcos MÁ. Función familiar y salud mental del cuidador de familiares con dependencia. *Aten Prim.* (2009) 41:621–8. 10.1016/j.aprim.2009.03.005 19497641 PMC7022046

[18] Rodriguez-Sanchez E Patino-Alonso MC Mora-Simón S Gómez-Marcos MA Pérez-Peñaranda A Losada-Baltar A Effects of a psychological intervention in a primary health care center for caregivers of dependent relatives: A randomized trial. *Gerontologist.* (2013) 53:397–406. 10.1093/geront/gns086 22899425

[19] Isik AT Soysal P Solmi M Veronese N. Bidirectional relationship between caregiver burden and neuropsychiatric symptoms in patients with Alzheimer’s disease: A narrative review. *Int J Geriatr Psychiatry.* (2019) 34:1326–34. 10.1002/gps.4965 30198597

[20] Zarit SH Reever KE Bach-Peterson J. Relatives of the impaired elderly: Correlates of feelings of burden. *Gerontologist.* (1980) 20:649–55. 10.1093/geront/20.6.649 7203086

[21] Narváes Bravo ML Martínez Martínez D. Caracterización del síndrome de sobrecarga del cuidador en familiares de pacientes institucionalizados y no institucionalizados con diagnóstico de enfermedad de Alzheimer mediante la escala Zarit (Characterization of caregiver burden syndrome). *Incl Desarro.* (2015) 3:101–7. 10.26620/uniminuto.inclusion.3.1.2016.101-107

[22] Aguilar Gutiérrez AE Jiménez Reyes J Álvarez Aguirre A Sánchez Perales M Ortega Jiménez M. Sobrecarga del cuidador principal del adulto mayor con enfermedad alzheimer. *Epistemus.* (2016) 10:30–6. 10.36790/epistemus.v10i21.31

[23] Galvis MJ Cerquera Córdoba AM. Relationship between depression and burden in caregivers of alzheimer disease patients. *Psicol Desde Caribe.* (2016) 33:83–103. 10.14482/psdc.33.2.6307

[24] Pearlin LI Mullan JT Semple SJ Skaff MM. Caregiving and the stress process: An overview of concepts and their measures. *Gerontologist.* (1990) 30:583–94. 10.1093/geront/30.5.583 2276631

[25] Savage S Bailey S. The impact of caring on caregivers’ mental health: A review of the literature. *Aust Health Rev.* (2004) 27:111. 10.1071/ah042710111 15362303

[26] Losada A Montorio I Izal M Márquez M. *Estudio e intervención sobre el malestar psicológico de los cuidadores de personas con demencia. El papel de los pensamientos disfuncionales.* Madrid: IMSERSO (2005).

[27] Turró O Soler O Garre J López S Vilalta J Monserrat S. Distribución factorial de la carga en cuidadores de pacientes con enfermedad de Alzheimer. *Rev Neurol.* (2008) 46:582–8. 18465696

[28] Crespo M Rivas MT. La evaluación de la carga del cuidador: Una revisión más allá de la escala de Zarit. *Clin Salud.* (2015) 26:9–16. 10.1016/j.clysa.2014.07.002

[29] Artaso B Goñi A Biurrun A. Cuidados informales en la demencia: Predicción de sobrecarga en cuidadoras familiares. *Rev Esp Geriatr Gerontol.* (2003) 38:212–8. 10.1016/s0211-139x(03)74886-0

[30] Blanco V Guisande MA Sánchez MT Otero P López L Vázquez FLS. Índrome de carga del cuidador y factores asociados en cuidadores familiares gallegos. *Rev Esp Geriatr Gerontol.* (2019) 54:19–26. 10.1016/j.regg.2018.03.005 30646994

[31] Bertel De la Hoz AM. Riesgo a enfermar y sobrecarga del cuidador principal del anciano dependiente. *Rev Cienc Bioméd.* (2020) 3:77–85. 10.32997/rcb-2012-3170

[32] Fundacio Pere Tarrés. *Proyectes Socials. La calidad de vida de las cuidadoras informales: Bases para un sistema de valoración. Informe II: El impacto del cuidado en la calidad de vida de las cuidadoras informales.* Madrid: Ministerio de trabajo e inmigración, Secretaria de Estado de Seguridad Social (2024).

[33] Ramón-Arbués E Martínez-Abadía B Martín-Gómez S. Factores determinantes de la sobrecarga del cuidador. Estudio de las diferencias de género. *Aten Prim.* (2017) 49:308–9. 10.1016/j.aprim.2016.07.003 28427914 PMC6876004

[34] Embracing Carers. *Informe sobre el bienestar de los cuidadores 2020. Quién cuida a los que cuidan? Así afectan la Covid-19 y la falta de apoyo a los cuidadores no profesionales.* Madrid: Merck (2021).

[35] Instituto Nacional de Estadística. *Encuesta de discapacidad, autonomía personal y situaciones de dependencia 2020. Cuidadores y cuidados. Cifras absolutas.* Madrid: Instituto Nacional de Estadística (2022).

[36] Belle SH. Enhancing the quality of life of dementia caregivers from different ethnic or racial groups: A randomized, controlled trial. *Ann Intern Med.* (2006) 145:727. 10.7326/0003-4819-145-10-200611210-00005 17116917 PMC2585490

[37] Márquez-González M Losada A Izal M Pérez-Rojo G Montorio I. Modification of dysfunctional thoughts about caregiving in dementia family caregivers: Description and outcomes of an intervention programme. *Aging Ment Health.* (2007) 11:616–25. 10.1080/13607860701368455 18074249

[38] Gallagher-Thompson D Coon DW. Evidence-based psychological treatments for distress in family caregivers of older adults. *Psychol Aging.* (2007) 22:37–51. 10.1037/0882-7974.22.1.37 17385981

[39] Saavedra Macías FJ Bascón Díaz MJ Árias Sánchez S García Calderón M Mora Moreno D. Cuidadoras de familiares dependientes y salud: Influencia de la participación en un taller de control de estrés. *Clin Salud.* (2013) 24:85–93. 10.5093/cl2013a10

[40] Arango-Lasprilla JC Panyavin I Merchán EJH Perrin PB Arroyo-Anlló EM Snipes DJ Evaluation of a group cognitive–behavioral dementia caregiver intervention in Latin America. *Am J Alzheimers Dis Other Dement.* (2014) 29:548–55. 10.1177/1533317514523668 24550547 PMC10852697

[41] Losada A Márquez-González M Romero-Moreno R. Mechanisms of action of a psychological intervention for dementia caregivers: Effects of behavioral activation and modification of dysfunctional thoughts. *Int J Geriatr Psychiatry.* (2011) 26:1119–27. 10.1002/gps.2648 21061414

[42] Losada A Márquez-González M Romero-Moreno R Mausbach BT López J Fernández-Fernández V Cognitive–behavioral therapy (CBT) versus acceptance and commitment therapy (ACT) for dementia family caregivers with significant depressive symptoms: Results of a randomized clinical trial. *J Consult Clin Psychol.* (2015) 83:760–72. 10.1037/ccp0000028 26075381

[43] Otero P Vázquez FL Ferraces MJ Blanco V Torres Á. Prevención de la depresión en cuidadoras no profesionales: Relación entre habilidades de solución de problemas y síntomas depresivos. *Clin Salud.* (2015) 26:1–7. 10.1016/j.clysa.2014.07.001

[44] Lewis ML Hobday JV Hepburn KW. Internet-based program for dementia caregivers. *Am J Alzheimers Dis Other Dement.* (2010) 25:674–9. 10.1177/1533317510385812 21131674 PMC10845646

[45] Boots LMM de Vugt ME Kempen GI Verhey FRJ. Effectiveness of a blended care self-management program for caregivers of people with early-stage dementia (partner in balance): Randomized controlled trial. *J Med Internet Res.* (2018) 20:e10017. 10.2196/10017 30006327 PMC6064039

[46] Cristancho-Lacroix V Wrobel J Cantegreil-Kallen I Dub T Rouquette A Rigaud A-SA. web-based psychoeducational program for informal caregivers of patients with Alzheimer’s disease: A pilot randomized controlled trial. *J Med Internet Res.* (2015) 17:e117. 10.2196/jmir.3717 25967983 PMC4468784

[47] Steffen AM Gant JRA. Telehealth behavioral coaching intervention for neurocognitive disorder family carers. *Int J Geriatr Psychiatry.* (2016) 31:195–203. 10.1002/gps.4312 26077904 PMC4744782

[48] Yepes-Nuñeza JJ Urrútiac G Romero-Garcíae M Alonso-Fernándeze S. Declaración PRISMA 2020: Una guía actualizada para la publicación de revisiones sistemáticas. *Rev Esp Cardiol.* (2021) 74:790–9. 10.1016/j.recesp.2021.06.016 34446261

[49] Moher D Liberati A Tetzlaff J Altman DG. Preferred reporting items for systematic reviews and meta-analyses: The PRISMA statement. *PLoS Med.* (2009) 6:e1000097. 10.1371/journal.pmed.1000097 19621072 PMC2707599

[50] Possin KL Merrilees JJ Dulaney S Bonasera SJ Chiong W Lee K Effect of collaborative dementia care via telephone and internet on quality of life, caregiver well-being, and health care use: The care ecosystem randomized clinical trial. *JAMA Intern Med.* (2019) 179:1658. 10.1001/jamainternmed.2019.4101 31566651 PMC6777227

[51] Meichsner F Theurer C Wilz G. Acceptance and treatment effects of an internet-delivered cognitive-behavioral intervention for family caregivers of people with dementia: A randomized-controlled trial. *J Clin Psychol.* (2019) 75:594–613. 10.1002/jclp.22739 30597537

[52] Cerquera-Córdoba AM Tiga-Loza DC Álvarez-Anaya WA Dugarte-Peña E Jaimes-Espíndola LR Plata-Osma LJ. Ensayo controlado aleatorizado de un programa multicomponente para cuidadores informales de pacientes con Alzheimer. *Rev Cuid.* (2021) 12:e2002. 10.15649/cuidarte.2002

[53] Birkenhäger-Gillesse EG Achterberg WP Janus SIM Kollen BJ Zuidema SU. Effects of caregiver dementia training in caregiver-patient dyads: A randomized controlled study. *Int J Geriatr Psychiatry.* (2020) 35:1376–84. 10.1002/gps.5378 32662184 PMC7689696

[54] Teles S Ferreira A Paúl C. Feasibility of an online training and support program for dementia carers: Results from a mixed-methods pilot randomized controlled trial. *BMC Geriatr.* (2022) 22:173. 10.1186/s12877-022-02831-z 35232389 PMC8887647

[55] Donath C Luttenberger K Graessel E Scheel J Pendergrass A Behrndt E-M. Can brief telephone interventions reduce caregiver burden and depression in caregivers of people with cognitive impairment? – Long-term results of the German day-care study (RCT). *BMC Geriatr.* (2019) 19:196. 10.1186/s12877-019-1207-y 31345170 PMC6659298

[56] Kor PPK Liu JYW Chien WT. Effects of a modified mindfulness-based cognitive therapy for family caregivers of people with dementia: A randomized clinical trial. *Gerontologist.* (2021) 61:977–90. 10.1093/geront/gnaa125 32886746

[57] Christie HL Dam AEH van Boxtel M Köhler S Verhey F de Vugt ME. Lessons learned from an effectiveness evaluation of in life, a web-based social support intervention for caregivers of people with dementia: Randomized controlled trial. *JMIR Aging.* (2022) 5:e38656. 10.2196/38656 36476485 PMC9773030

[58] Dichter MN Albers B Trutschel D Ströbel AM Seismann-Petersen S Wermke K TALKING TIME: A pilot randomized controlled trial investigating social support for informal caregivers via the telephone. *BMC Health Serv Res.* (2020) 20:788. 10.1186/s12913-020-05523-9 32838773 PMC7446183

[59] Tinoco-Camarena JM Puig-Llobet M Lluch-Canut MT Roldan-Merino J Moreno-Arroyo MC Moreno-Poyato A Effectiveness of the online “dialogue circles” nursing intervention to increase positive mental health and reduce the burden of caregivers of patients with complex chronic conditions. Randomized clinical trial. *Int J Environ Res Public Health.* (2022) 20:644. 10.3390/ijerph20010644 36612964 PMC9819240

[60] Sanjuán-Quiles A Alcañiz-Garrán MM Montejano-Lozoya R Ramos-Pichardo JD García-Sanjuán S. La perspectiva de las personas cuidadoras desde un análisis de género. *Rev Esp Salud Publica.* (2023) 97:e202307062. PMC10541258 37415488

[61] Losada A Izal M Montorio I Márquez M Pérez G. Eficacia diferencial de dos intervenciones psicoeducativas para cuidadores de familiares con demencia. *Rev Neurol.* (2004) 38:701–8. 15122537

[62] Losada A Moreno-Rodríguez R Cigarán M Peñacoba C Montorio I. Análisis de programas de intervención psicosocial en cuidadores de pacientes con demencia. *Inform Psiquiatr.* (2006) 184:173–86.

[63] Kajiyama B Thompson LW Eto-Iwase T Yamashita M Di Mario J Marian Tzuang Y Exploring the effectiveness of an Internet-based program for reducing caregiver distress using the iCare stress management e-training program. *Aging Ment Health.* (2013) 17:544–54. 10.1080/13607863.2013.775641 23461355 PMC3695021

[64] Cheng S-T Li K-K Losada A Zhang F Au A Thompson LW The effectiveness of nonpharmacological interventions for informal dementia caregivers: An updated systematic review and meta-analysis. *Psychol Aging.* (2020) 35:55–77. 10.1037/pag0000401 31985249

[65] Acton GJ Kang J. Interventions to reduce the burden of caregiving for an adult with dementia: A meta-analysis§. *Res Nurs Health.* (2001) 24:349–60. 10.1002/nur.1036 11746065

[66] Kwon O-Y Ahn HS Kim HJ Park K-W. Effectiveness of cognitive behavioral therapy for caregivers of people with dementia: A systematic review and meta-analysis. *J Clin Neurol.* (2017) 13:394. 10.3988/jcn.2017.13.4.394 29057632 PMC5653628

[67] Bustillo ML Gómez-Gutiérrez M Guillén AI. Los cuidadores informales de personas mayores dependientes: Una revisión de las intervenciones psicológicas de los últimos diez años. *Clin Salud.* (2018) 29:89–100. 10.5093/clysa2018a13

[68] Collins RN Kishita N. The effectiveness of mindfulness- and acceptance-based interventions for informal caregivers of people with dementia: A meta-analysis. *Gerontologist.* (2019) 59:e363–79. 10.1093/geront/gny024 29635303

[69] Livingston G Barber J Rapaport P Knapp M Griffin M King D Clinical effectiveness of a manual based coping strategy programme (START, STrAtegies for RelaTives) in promoting the mental health of carers of family members with dementia: Pragmatic randomised controlled trial. *BMJ.* (2013) 347:f6276–6276. 10.1136/bmj.f6276 24162942 PMC3808082

[70] Wilz G Soellner R. Evaluation of a short-term telephone-based cognitive behavioral intervention for dementia family caregivers. *Clin Gerontol.* (2016) 39:25–47. 10.1080/07317115.2015.1101631

[71] Drentea P Clay OJ Roth DL Mittelman MS. Predictors of improvement in social support: Five-year effects of a structured intervention for caregivers of spouses with Alzheimer’s disease. *Soc Sci Med.* (2006) 63:957–67. 10.1016/j.socscimed.2006.02.020 16616406

[72] Roth DL Mittelman MS Clay OJ Madan A Haley WE. Changes in social support as mediators of the impact of a psychosocial intervention for spouse caregivers of persons with Alzheimer’s disease. *Psychol Aging.* (2005) 20:634–44. 10.1037/0882-7974.20.4.634 16420138

